# Aldehyde-specific responses of olfactory sensory neurons in the praying mantis

**DOI:** 10.1038/s41598-021-81359-5

**Published:** 2021-01-20

**Authors:** Kota Ezaki, Takashi Yamashita, Thomas Carle, Hidehiro Watanabe, Fumio Yokohari, Yoshifumi Yamawaki

**Affiliations:** 1 Department of Biology, Faculty of Science, Kyushu University, Fukuoka, 819-0395 Japan; 2 Department of Earth System Science, Faculty of Science, Fukuoka University, Fukuoka, Japan

**Keywords:** Olfactory system, Neurophysiology

## Abstract

Although praying mantises rely mainly on vision for predatory behaviours, olfaction also plays a critical role in feeding and mating behaviours. However, the receptive processes underlying olfactory signals remain unclear. Here, we identified olfactory sensory neurons (OSNs) that are highly tuned to detect aldehydes in the mantis *Tenodera aridifolia*. In extracellular recordings from OSNs in basiconic sensilla on the antennae, we observed three different spike shapes, indicating that at least three OSNs are housed in a single basiconic sensillum. Unexpectedly, one of the three OSNs exhibited strong excitatory responses to a set of aldehydes. Based on the similarities of the response spectra to 15 different aldehydes, the aldehyde-specific OSNs were classified into three classes: B, S, and M. Class B broadly responded to most aldehydes used as stimulants; class S responded to short-chain aldehydes (C3–C7); and class M responded to middle-length chain aldehydes (C6–C9). Thus, aldehyde molecules can be finely discriminated based on the activity patterns of a population of OSNs. Because many insects emit aldehydes for pheromonal communication, mantises might use aldehydes as olfactory cues for locating prey habitat.

## Introduction

Olfaction plays a critically important role in the behaviours of not only phytophagous and polyphagous insects but also entomophagous insects. The adults of the damselfly *Ischnura elegans*, for example, are attracted to odours emitted by prey^1^. Olfactory cues are also potentially involved in sex recognition in *I. elegans*^2^. The aphid predator *Aphidoletes aphidimyza* is attracted to the phenylacetaldehyde odour of the honeydew excreted by aphids *Aphis gossypii*^3^. Moreover, olfaction is more important than vision for several species of assassin bugs during the initial stage of prey detection^4^. The central nervous system of entomophagous insects likely integrates multimodal information, including visual and olfactory cues, for locating their prey.

The main olfactory organs of insects are olfactory sensilla on antennae. Each olfactory sensillum has several olfactory sensory neurons (OSNs) that often have different response characteristics. OSNs extend their axons into the primary olfactory centre in the brain, the antennal lobe, which is compartmentalized into spherical neuropils called glomeruli. In most insects, each glomerulus receives sensory input from a single type of OSN that expresses a cognate odourant receptor^5^. Hence, the number of glomeruli is almost equal to the number of different types of OSNs and is possibly related to the capacity for odour discrimination. The olfactory sensilla, OSNs, and the functional organization of the antennal lobe have been studied in many phytophagous and polyphagous insects^5–7^. However, little is known of the olfactory systems of entomophagous insects, with the exception of dragonflies and damselflies^1,8–10^.

The praying mantis is an entomophagous insect that shows both visual and olfactory guided-behaviours, but its olfactory system has been poorly studied. In several species of mantis, sex pheromones emitted from females attract males^11–14^. The mantis *Sphodromantis lineola* eats a diced banana after drumming it with the antennae, and this feeding behaviour can be elicited by banana odours^15^. The antenna of the mantis *Tenodera aridifolia* possesses six types of sensilla: basiconic, trichoid, grooved peg, chaetic, campaniform, and coelocapitular sensilla^16^. Basiconic, trichoid, and grooved peg sensilla are considered olfactory, and each type of sensilla likely plays a different role in coding a variety of odours. The grooved peg sensilla are presumably involved in sex pheromone detection because sensilla on the antenna are numerous in adult males but there are few in females and nymphae^12,16,17^. However, the functional roles of other olfactory sensilla remain unknown.

Here, we studied the basiconic sensilla on the antennae of *T. aridifolia*, which are potentially involved in food detection. OSNs responding to food-related odours have been found in the basiconic sensilla of several insect species^18–20^. The longhorned beetle *Monochamus galloprovincialis*, for example, has OSNs in basiconic sensilla that respond to host plant volatiles^19^. Basiconic sensilla in a blood-sucking bug *Triatoma infestans* are excited by host odours^18^. To understand the functional role of basiconic sensilla in mantises, we studied the response properties of OSNs using single-sensillum recording. Unexpectedly, we found that some basiconic OSNs specifically responded to aldehyde odours.

## Results

### Three olfactory sensory neurons (OSNs) in basiconic sensilla

**Figure 1:**
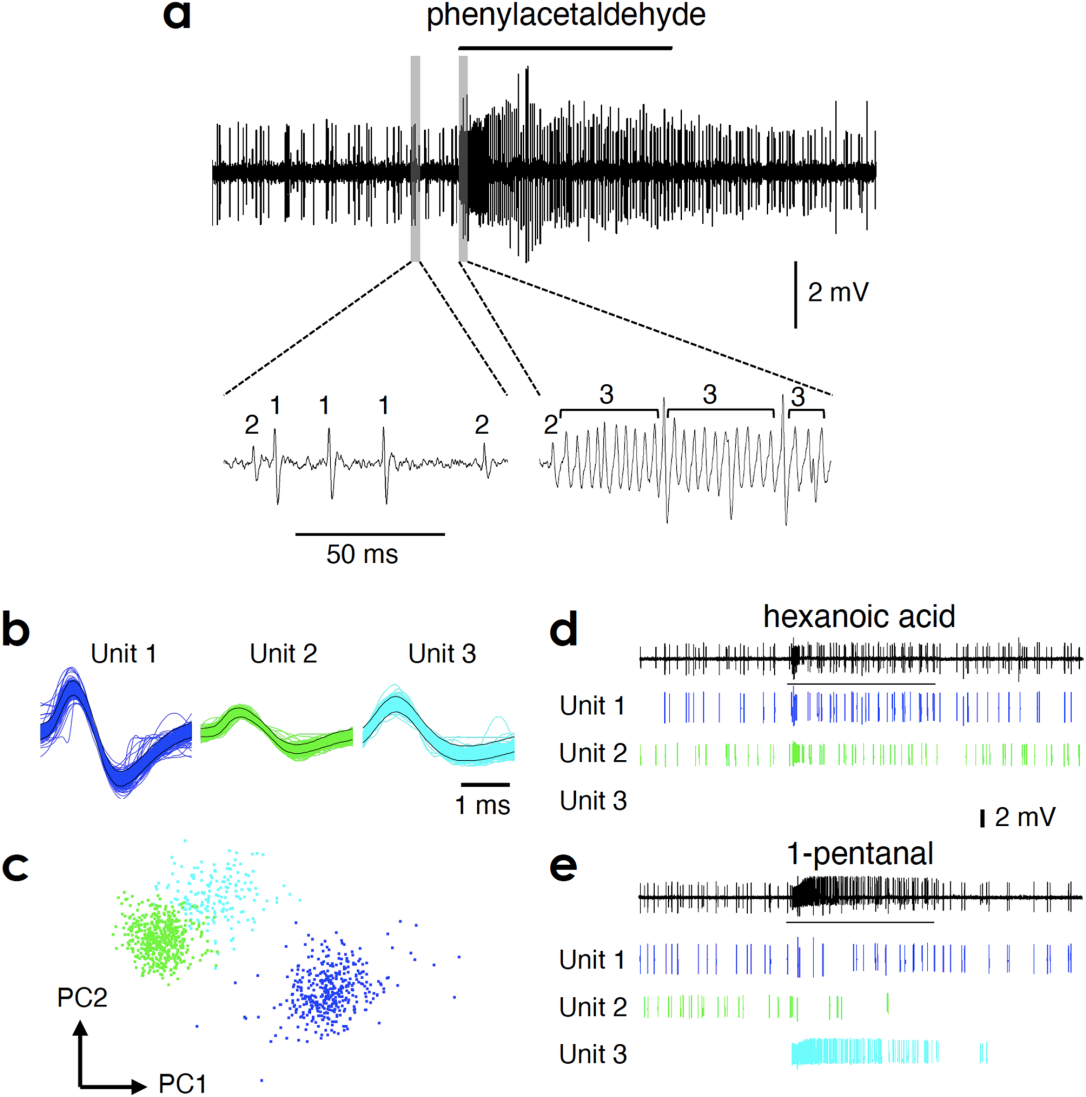
Identification of three olfactory sensory neurons (OSNs) in a single basiconic sensillum. (**a**) Top trace indicates a sample recording from a single basiconic sensillum. The black bar above the recording indicates the period of odour stimulation (2 s). The expanded traces are shown at the bottom. Three different shapes of action potentials (spikes) were observed and are referred to as unit 1, 2, and 3. Unit 1 and 2 showed spontaneous firing, but unit 3 did not. Letters above spikes indicate the unit number. (**b**) The spike waveforms of unit 1 (blue), 2 (green), and 3 (sky blue) are superimposed. Black traces indicate the templates used for sorting spikes. Data are from another sample recording. (**c**) Each spike of unit 1–3 (shown in b) is plotted based on the first two principal components (PC1 and PC2) obtained from the principal component analysis. (**d**,**e**) Sample responses of unit 1, 2, and 3 to hexanoic acid (**d**) and 1-pentanal (**e**). Top traces indicate recordings from the same basiconic sensillum. Black bars under the recordings indicate the period of odour stimulation (2 s). Spikes of each unit are separately indicated under the recording.

Responses of OSNs to odours were extracellularly recorded by inserting a sharp-pointed tungsten electrode into the base of single sensillum (single-sensillum recordings, SSRs). Because basiconic sensilla of *T. aridifolia* have not been classified into types based on their shapes and positions, we randomly selected a sensillum on the antenna. In most recordings from single basiconic sensilla, three different shapes of action potentials (spikes) were observed (Fig. 1). Using spike sorting software based on the spike shape, we successfully sorted spikes into three types (Fig. 1b), which was confirmed by principal component analysis (Fig. 1c). We concluded that these spikes reflected the activities of three OSNs housed in single basiconic sensillum. Two types of spikes showed spontaneous activities, and spikes with larger and smaller amplitudes were termed unit 1 and 2, respectively (Fig. 1a). The other type of spikes that did not show spontaneous firing was termed unit 3 (Fig. 1a). Unit 1 and 2 showed excitatory (Fig. 1d) or inhibitory (Fig. 1e) responses to many types of odours. Generally, unit 1 and 2 exhibited on-phasic excitatory responses to effective odours. Unit 3 showed strong excitatory phasic-tonic responses to effective odours, which often continued beyond the end of odour presentation (Fig. 1e).

### Basiconic OSNs tuned to aldehydes

**Figure 2:**
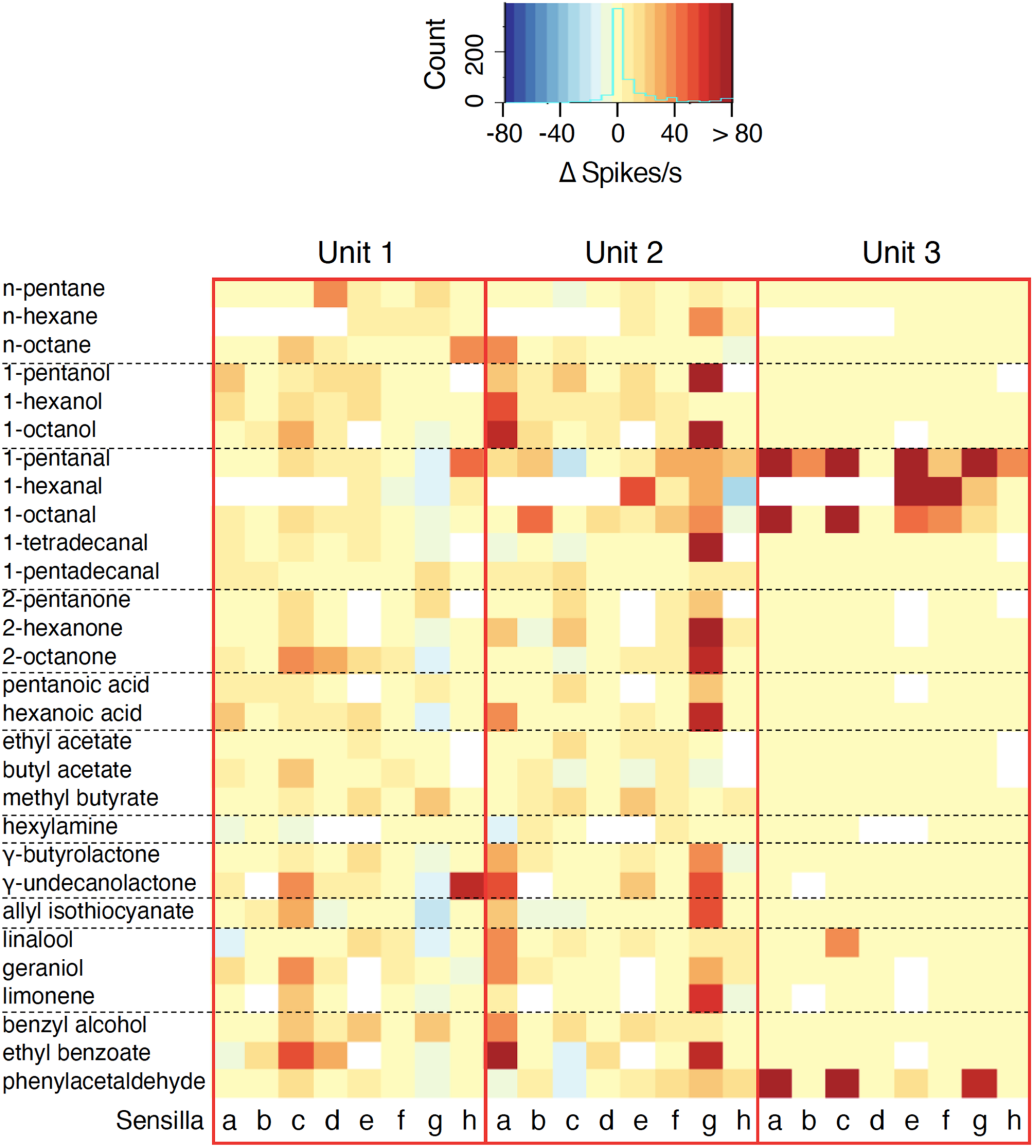
A heat map of the response intensities of a single OSN to tested odours covering a wide range of functional groups. Each column indicates a single OSN showing unit 1 (left), 2 (centre), and 3 (right) type spikes in recordings. Response intensity was defined as the spike frequency (Hz) during 2 s after the onset of odour stimulation subtracted by that during 2 s before the onset of stimulation; and it is shown in pseudo-colour. White colour indicates that no recording was made. Letters under columns indicate individual sensilla. Odours with failed recordings from most sensilla were excluded from the analysis. The heat map was made using R 3.6.1 software (https://www.r-project.org/).

To characterize the odour response properties of triad OSNs in the single basiconic sensilla, we presented a set of 33 types of commercially available odourants (Table 1). Odours were selected to represent a wide range of functional groups (alkanes, alcohols, aldehydes, ketones, carboxylic acids, esters, amines, lactones, sulphide, terpenes, and aromatics), and selected chemicals were dissolved at 10^−1^ dilution with solvents (ethanol, *n*-hexane, or diethyl phthalate). Each OSN showed either few or no responses to solvent odours. Among 57 sensilla tested, we analysed data from eight sensilla because responses to more than 20 odours were recorded from each of these sensilla (See Supplementary Fig. S1 online). While most OSNs of unit 1 and 2 showed broad excitatory responses to the odours of several functional groups, most OSNs of unit 3 responded narrowly to specific odours, such as 1-pentanal, 1-hexanal, 1-octanal, and phenylacetaldehyde (Fig. 2). Although we initially classified it as an aromatic, phenylacetaldehyde also has an aldehyde group as a functional moiety. Hence, we concluded that unit 3 OSNs were narrowly tuned to aldehyde odours.

**Table 1:** Compounds used for characterizing general responses.

Functional group	Compound	Purity (%)	Solvent		Source
Alkane	*n*-Pentane	98	Ethanol		WPC
	*n*-Hexane	96	Ethanol		WPC
	*n*-Octane	98	Ethanol		WPC
Alcohol	1-Pentanol	98	Ethanol		WPC
	1-Hexanol	97	Ethanol		WPC
	1-Octanol	98	Ethanol		WPC
Aldehyde	1-Pentanal	98	Ethanol		KC
	1-Hexanal	95	Ethanol		WPC
	1-Octanal	97	Ethanol		WPC
	1-Tetradecanal	95	*n*-Hexane		CS
	1-Pentadecanal	97	Ethanol		AC
Ketone	2-Pentanone	95	Ethanol		WPC
	2-Hexanone	95	Ethanol		WPC
	2-Octanone	97	Ethanol		KC
Carboxylic acid	Pentanoic acid	95	Ethanol		WPC
	Hexanoic acid	99	Ethanol		WPC
	Octanoic acid	96	Ethanol		SA
Ester	Ethyl acetate	99	Ethanol		WPC
	Butyl acetate	99	Ethanol		TCI
	Methyl *n*-butyrate	98	Ethanol		WPC
Amine	Hexylamine	99	Ethanol		TCI
	Trimethylamine	30	Ethanol		WPC
Lactone	γ-Butyrolactone	99	Ethanol		TCI
	γ-Undecanolactone	98	Ethanol		TCI
Sulfide	Allyl isothiocyanate	92	Ethanol		TCI
Terpene	Linalool	98	Ethanol		WPC
	Geraniol	97	Ethanol		WPC
	Limonene	95	Ethanol		WPC
Aromatic	Benzyl alcohol	99	Ethanol		WPC
	Ethyl benzoate	98	Ethanol		WPC
	Benzaldehyde	98	Ethanol		WPC
	Phenylacetaldehyde	40–60	Ethanol	Diethyl phthalate	WPC
	Maltol	99	Ethanol		WPC

*TCI* Tokyo Chemical Industry, *KC* Kanto Chemical, *SA* Sigma-Aldrich, *AC* Angene Chemical, *CS* ChemSampCo, *WPC* Wako Pure Chemical.

### Differences in specificity to aldehydes

**Figure 3:**
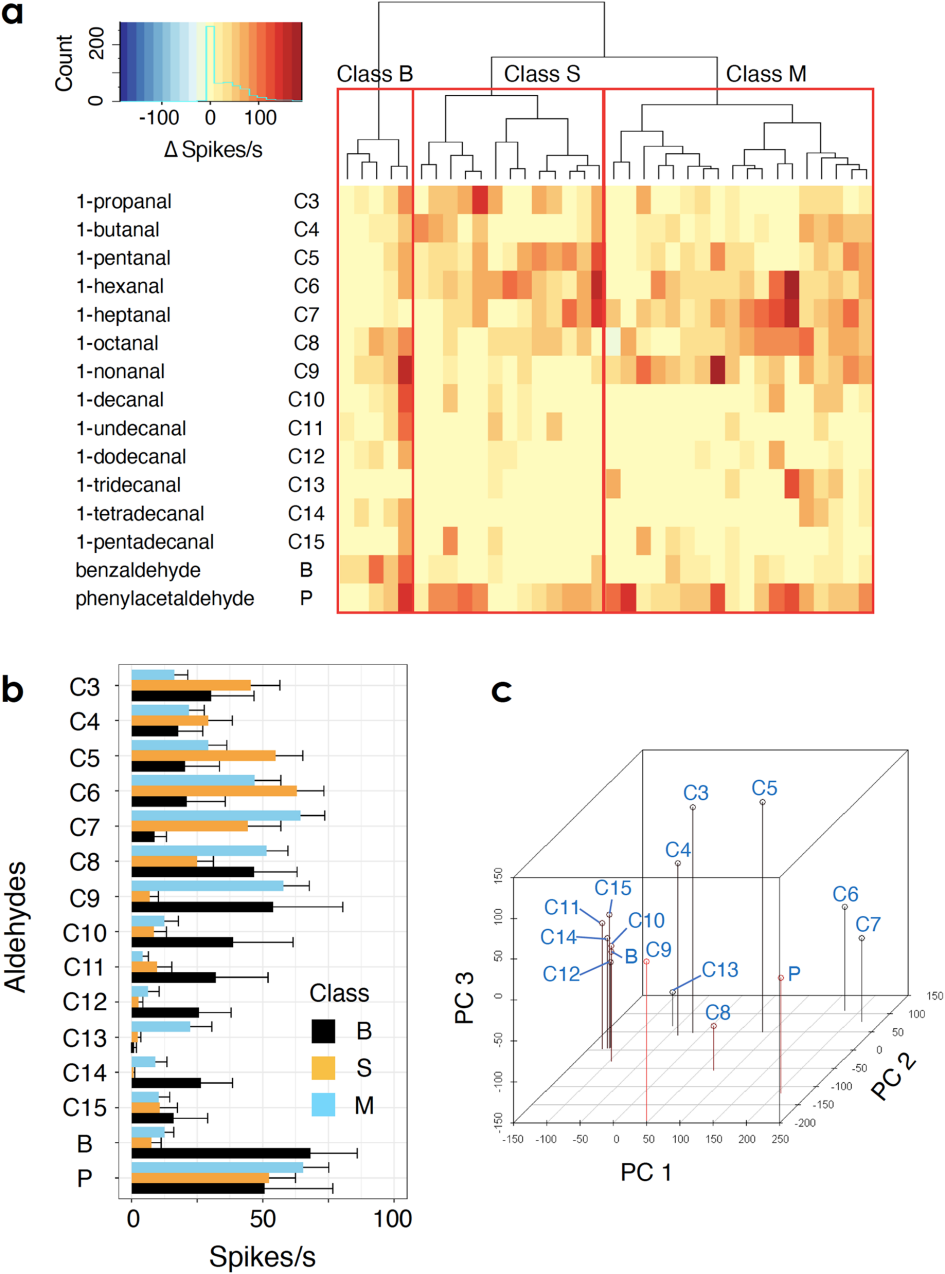
(**a**) A heat map of the response intensities of a single OSN to tested aldehydes. Each column indicates a single OSN showing a unit 3-type spike. A dendrogram of a hierarchical cluster analysis (shown above the heat map) suggested three classes: B, S, and M. Class B OSNs broadly responded to most aldehydes, including benzaldehyde. Class S and M responded strongly to short (C3–C7) and middle-length chain (C6–C9) aldehydes, respectively. The distance was defined as 1 − *r* (correlation coefficient). (**b**) Mean responses of classes B (black), S (orange), and M (sky blue) to aldehyde odours. Error bars indicate standard error. See the heat map (**a**) for abbreviations of aldehydes. **c** The first three principal components (PC1–3) of an OSN space that correlate aldehydes with the OSNs that they activate. R 3.6.1 software (https://www.r-project.org/) was used for analysis.

To further characterize the aldehyde-specific responses of unit 3 OSNs, we presented a set of 15 aldehydes to the basiconic sensillum (Table 2). Among 106 sensilla tested, we analysed data from 36 sensilla because responses to all 15 aldehydes were recorded from each of these sensilla (see Supplementary Fig. S2 online). We pooled data from males and females because there was no significant effect of sex (ANOVA, *n* = 540, *p* = 0.968). A hierarchical cluster analysis and heatmap of OSN responses indicated that the 36 OSNs could be classified into three functional classes: class B, S, and M (Fig. 3). In this study, a class did not correspond to a specific OSN; instead, classes contained several different OSNs showing a similar response pattern. This classification helped differentiate the OSN population. Class B OSNs showed broad responses to most aldehydes, including benzaldehyde (Fig. 3a,b). Classes S and M responded most strongly to short (C3–C7) and middle-length chain (C6–C9) aldehydes, respectively (Fig. 3a,b). Classes S and M responded weakly to benzaldehyde. All of these classes showed little or no responses to 1-tetradecanal and 1-pentadecanal. These two aldehydes have been identified as sex pheromones in the mantis *S. lineola*^11^.

**Table 2:** Compounds used for analyzing responses to aldehyde.

Compound	Purity (%)	Solvent		Source
1-Propanal	98	Ethanol		TCI
1-Butanal	98	Ethanol		WPC
1-Pentanal	98	Ethanol	Paraffin oil	KC
1-Hexanal	95	Ethanol		WPC
1-Heptanal	95	Ethanol		WPC
1-Octanal	97	Ethanol		WPC
1-Nonanal	95	Ethanol	Paraffin oil	WPC
1-Decanal	85	Ethanol		WPC
1-Undecanal	85	Ethanol		WPC
1-Dodecanal	85	*n*-Hexane		WPC
1-Tridecanal	96	*n*-Hexane		AA
1-Tetradecanal	95	*n*-Hexane		CS
1-Pentadecanal	97	Ethanol		AC
Benzaldehyde	98	Ethanol		WPC
Phenylacetaldehyde	40–60	Ethanol	Diethyl phthalate	WPC

*AA* Alfa Aesar, *AC* Angene Chemical, *CS* ChemSampCo, *KC* Kanto Chemical, *TCI* Tokyo Chemical Industry, *WPC* Wako Pure Chemical.

Thus, most unit 3 OSNs respond to only subsets of aldehydes, and individual aldehydes activate only small subsets of unit 3 OSNs. Next, we constructed an ‘odour receptive space’ that correlates aldehydes with the OSNs that they activate, where each axis represents the responses of one OSN. Using principal component analysis, we transformed this into a three-dimensional space represented by the first three principal components (Fig. 3c). In this space, aldehydes that elicited similar activity patterns of OSNs were mapped closer together. The short and middle-length chain aldehydes (C3–C9) and phenylacetaldehyde (P) were well separated in this space, suggesting that mantises can discriminate these aldehydes based on the combinatorial activity patterns of unit 3 OSNs. In contrast, benzaldehyde (B) and the long-chain aldehydes (C10–C15), except 1-tridecanal, clustered together.

### Dose responses to aldehydes

Our results indicated that there were three classes of unit 3 OSNs and that the specificity to aldehydes differed among classes. However, the differences in sensitivity among classes remained unclear. Finally, we examined the sensitivity of unit 3 OSNs to aldehydes. We recorded responses from 44 sensilla and analysed data of 10 sensilla to four different concentrations of 1-pentanal, 1-nonanal, and phenylacetaldehyde. 1-pentanal and 1-nonanal elicited strong responses by class S and M, respectively, while phenylacetaldehyde broadly activated all classes. Responses to 1-propanal, 1-heptanal, 1-tridecanal, and benzaldehyde were also recorded so that OSNs could be classified based on their responses to these aldehydes.

**Figure 4:**
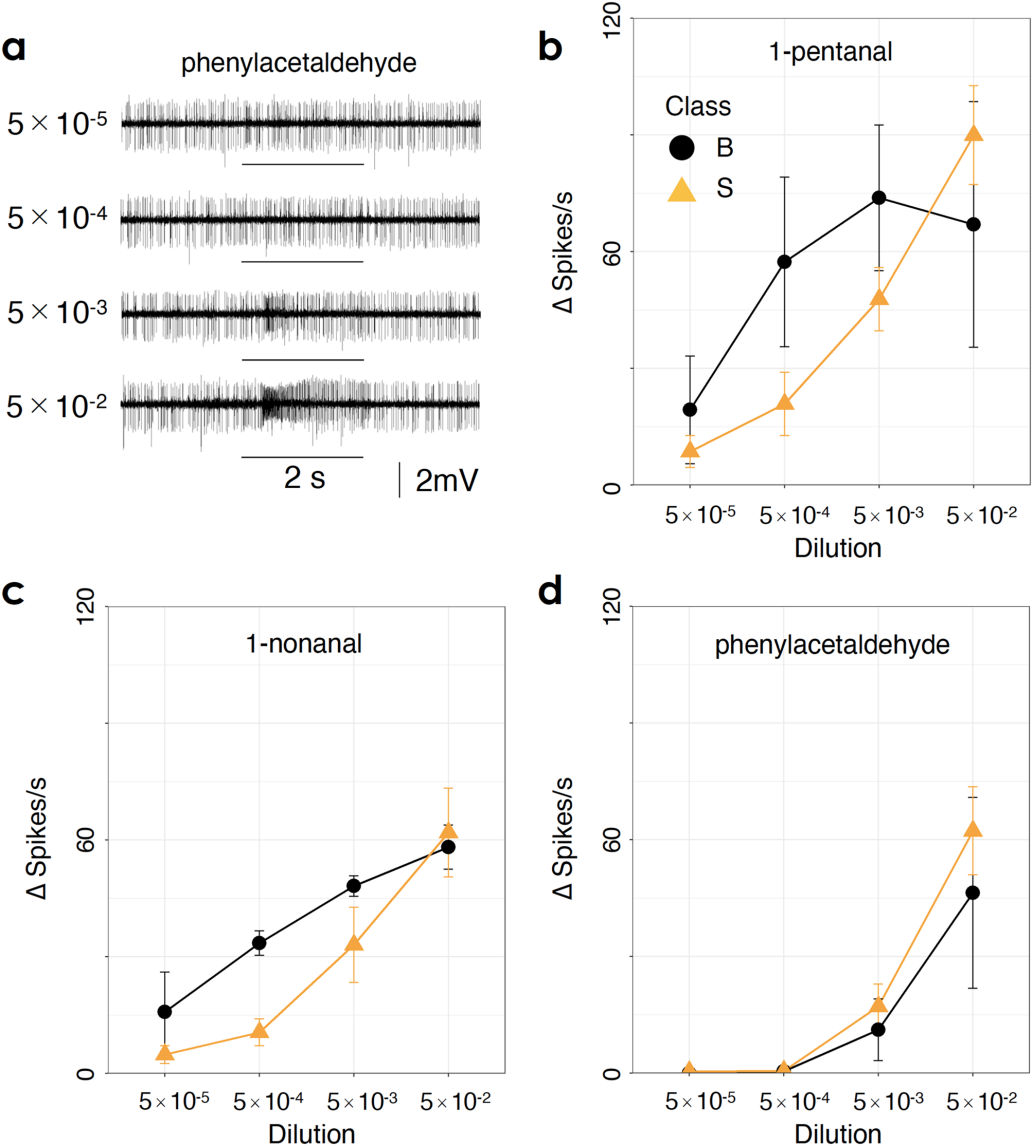
(**a**) Sample responses to phenylacetaldehyde at dilutions from 5 × 10^−5^ to 5 × 10^−2^. Sample responses were recorded from the same basiconic sensillum. Black bars under recordings indicate the period of odour stimulation (2 s). (**b**–**d**) Dose–response curves for OSNs (unit 3) to 1-pentanal (**b**), 1-nonanal (**c**), and phenylacetaldehyde (**d**). Responses to aldehydes suggested that seven OSNs belonged to class S (orange triangle) and that three OSNs belonged to class B (black circle). The mean response and standard error for each class are plotted against the dilution.

The spike frequency of unit 3 OSNs generally increased as the concentrations of each odour increased (Fig. 4). Additionally, the temporal response pattern also changed: strong firing continued for longer periods at higher concentrations (Fig. 4a). Hierarchical cluster analysis suggested that 10 of the unit 3 OSNs analysed were classified into two groups (see Supplementary Fig. S3 online). One group (n = 7) showed stronger responses to 1-pentanal than to 1-nonanal and weak responses to benzaldehyde, suggesting that this group belonged to class S. The other group (n = 3) responded to 1-pentanal, 1-nonanal, and benzaldehyde at similar spike rates, suggesting that this group belonged to class B. ANOVA with all data revealed that odour and concentration had significant effects on OSN responses (*n* = 120; *p* < 0.001 for each of odour and concentration). Moreover, there was a significant interaction between class and concentration (*p* = 0.0136), suggesting that sensitivity differed between classes. The OSNs of class B appeared to have higher sensitivity to 1-pentanal (Fig. 4b) and 1-nonanal (Fig. 4c) than the OSNs of class S. However, sensitivity to phenylacetaldehyde differed little between class B and class S OSNs (Fig. 4d).

## Discussion

In this study, we performed single-sensillum recording from the antennal basiconic sensilla of the praying mantis *T. aridifolia*. This is the first study to characterize the specific features of peripheral olfactory reception in a mantis. We found that one of the OSNs in single basiconic sensilla was specifically tuned to aldehyde odours. The aldehyde-specific OSNs were classified into three classes: class B, which showed broad responses to most aldehydes presented; the class S, which responded to short-chain aldehydes; and class M, which responded to middle-length chain aldehydes (Fig. 3). Class B appeared to have higher sensitivity and lower specificity to aldehydes than the other classes (Figs. 3 and 4). Because the behaviours of mantises are known to rely heavily on their excellent vision, high specificity and sensitivity to aldehyde odours were unexpected. These results suggested that aldehyde and other odours might be separately processed in the peripheral sensory system and that the quality of each aldehyde molecule might be encoded by the activity patterns of a population of specific OSNs.

We concluded that a basiconic sensillum contained at least three OSNs because we observed three different shapes of spikes in the extracellular recordings from a single sensillum. Although our previous observations with transmission electron microscopy (TEM) revealed the presence of two sensory neurons in basiconic sensilla^16^, more than two sensory neurons might be present in most types of basiconic sensilla. In a few recordings, we observed only two different shapes of spikes. Hence, there might be several different types of basiconic sensilla: some with two sensory neurons and others with three or more sensory neurons. Detailed observations of the shapes and positions of sensilla, combined with the identification of chemoreceptor genes, are required to classify basiconic sensilla into several different types, followed by additional observations with TEM to confirm the number of OSNs housed in each type of basiconic sensilla.

The perception of aldehydes emitted from their prey is likely important for entomophagous insects. Aldehydes are contained in ‘green leaf volatiles’^21^ and the pheromones in many insect species^22–27^. In the locust *Schistocerca gregaria*, for example, hexanal, octanal, nonanal, and decanal have been documented in the aggregation pheromone emitted from nymphs^22^. In the beetle *Pachnoda interrupta*, phenylacetaldehyde was recorded in female volatile emissions and attracts both male and female conspecifics^25^. Moreover, phenylacetaldehyde is present in the honeydew excreted by the aphids *A. gossypii* and attracts the aphid predator *A. aphidimyza*^3^. Thus, mantises might also detect aldehydes to locate prey habitat. Aldehydes might also be used to discriminate live prey from dead prey because the degeneration of unsaturated cuticular hydrocarbons on insect integument generates long-chain aldehydes such as tetradecanal^28^.

However, the perception of aldehyde odours might also facilitate the evasion of vertebrate predators, such as lizards and birds^10^. Several hematophagous insects detect aldehydes emitted from their vertebrate hosts^18,29,30^. Nonanal, for example, is one of the major components of avian and human odours and attracts *Culex* mosquitoes^29^. Although mantises can visually detect predators^31^, the avoidance of habitats with predator odours could also provide an effective defence mechanism. To test these possibilities, behavioural analyses of the preferences of mantises for different odours are needed.

Although OSNs responding to aldehydes have been reported in many insects^10,18,20,29,30,32,33^, OSNs that are specific to aldehydes represent a small subset of them. In the cockroach *Periplaneta americana*, for example, OSNs responding to aldehydes (pentanal, hexanal, and heptanal) are also activated by alcohols (pentanol, hexanol, and heptanol)^33^. The ab4A OSN in *Drosophila* strongly responds to both E2-hexenal and E2-hexenol^20^. In contrast, the blood-sucking bug *T. infestans* has nonanal-specific OSNs in basiconic sensilla: they do not respond to nonane, nonanol, or nonanoic acid^18^. Similarly, mantis basiconic OSNs that respond to pentanal did not respond to pentane, pentanol, 2-pentanone, or pentanoic acid (Fig. 2). In hematophagous and entomophagous insects, aldehyde-specific OSNs might play an important role in detecting hosts or prey. Piersanti et al.^10^ reported that the dragonfly and damselfly OSNs respond to aldehydes, but they did not analyse the responses of single OSN to a set of odours from various functional groups. Thus, whether the OSNs of dragonflies and damselflies are specific or just responsive to aldehydes remains unclear. Aldehyde-specific OSNs have been found in several insect orders that are distantly related to mantises but not in closely related insect orders, such as cockroaches, suggesting that aldehyde specificity might have evolved in several insect orders independently.

Whether aldehyde-specific OSNs in mantises express odourant receptors (OR)^34^ or ionotropic receptors (IR)^35,36^ remains unclear. In insects, two distinct populations of OSNs expressing different chemoreceptor gene families have been identified: one group expresses OR family and the other expresses IR family. Insect olfactory sensilla are commonly classified into single-walled and double-walled types based on the hair wall structures^37^. OSNs expressing OR are housed in single-walled sensilla, such as basiconic and trichoid sensilla in the fruitfly^34^, whereas OSNs expressing IR are housed in double-walled sensilla, such as coeloconic sensilla in the fruitfly^36^. The basiconic sensilla in mantises have single-walled cuticular apparatuses^16^, suggesting that their OSNs express OR. However, IR show superior detection of aldehydes as aldehydes are one of several known strong IR ligands^35^. Thus, the receptor proteins expressed in mantis OSNs need to be identified.

IR are likely expressed in the OSNs of grooved peg sensilla in mantises, which have double-walled cuticular apparatuses with spoke canals^16^. The grooved peg sensilla are presumably involved in the detection of sex pheromones because adult males possess a large number of them on the antennae^12,16^. Sex pheromones identified from another mantis species, *S. lineola*, includes long-chain aldehydes, 1-tetradecanal, and 1-pentadecanal^11^. These aldehydes elicited little excitation of basiconic OSNs in this study. A long-chain aldehyde (triacontanal) is also found in the cuticular extracts of mantises^38^ but not in orthopteran insects, suggesting that it has a pheromonal function. In mantises, long-chain aldehydes are likely used in pheromonal communication and are detected by the grooved peg sensilla. However, the behavioural function of double-walled sensilla appears to differ among insect species. In the cockroach *P. americana*, for example, OSNs in the grooved, double-walled sensilla mainly respond to aldehydes and carboxylic acids^39^, but not to sex pheromones. Instead, two major components of sex pheromones of *P. americana* are detected by OSNs in the single-walled sensilla that have similar morphological features to the basiconic sensilla in mantises. Although cockroaches are closely related to mantises, the behavioural role of the sensilla do not appear to be conserved between these groups.

Olfactory sensilla in mantises have been morphologically classified^16^ and organization of the primary olfactory centre (antennal lobe) in the mantis brain has been studied^40^. However, the pathway of olfactory information, such as the projection pattern of OSNs to the antennal lobe, has not been well studied. Additional studies on the olfactory system in mantises would provide insight into mechanisms underlying the adaptation of the insect olfactory system in different insect orders during the evolutionary process.

## Methods

### Insects

We used adults of male and female mantises of *T. aridifolia*. The mantises were bred from ootheca collected in the suburb of Fukuoka, Japan. The nymphs were bred by previously described methods^41^.

### Odour stimuli

We used a set of 33 kinds of commercially available odourants to characterize the responses of the OSNs (Table 1). Odours were selected to represent many functional groups (alkanes, alcohols, aldehydes, ketones, carboxylic acids, esters, amines, lactones, sulphides, terpenes, and aromatics). 1-tetradecanal and 1-pentadecanal were also selected because these are the components of sex pheromones in another mantis species, *S. lineola*^11^. To examine the specificity to aldehydes, a set of 15 aldehydes was used (Table 2). Most odourants were dissolved by a 10^−1^ dilution (volume/volume for liquids, weight/weight for solids) in ethanol, but 1-dodecanal, 1-tridecanal, and 1-tetradecanal were diluted with *n*-hexane because these were not soluble in ethanol.

To study the effects of concentration on OSN responses, 1-pentanal, 1-nonanal, and phenylacetaldehyde were diluted stepwise, from 5 × 10^−5^ to 5 × 10^−2^. 1-propanal, 1-heptanal, 1-tridecanal, and benzaldehyde (5 × 10^−2^ for each) were also presented for classification of OSNs. To avoid changing the concentrations of solutions, odourants (except phenylacetaldehyde) were dissolved in paraffin oil, which was hard to volatilize. Phenylacetaldehyde was dissolved in diethyl phthalate because it was not soluble in paraffin oil.

### Odour presentation

Each odour was presented through an apparatus made following the methods of previous studies^42,43^. Fresh air was taken from outdoors by a pump, filtered with activated charcoal, and dehumidified with silica gel. The airflow was maintained at approximately 1 L/min using a flowmeter (RK1600R, KOFLOC) and passed through silicone tubes and a glass pipette, from which the odour was presented to the mantis. The pipette contained 20 µL of odour solution absorbed onto a small piece of filter paper (1.8 × 0.7 cm^2^). Between odour presentations, the fresh air continued to vent through other pipettes. Air around the preparation was continuously exhausted by a duct located behind the base of the antenna. The timing of odour presentation was controlled by a stimulator (SEN7203, Nihon Kohden) via a three-way solenoid valve. The stimulus duration was 2 s, and the inter-stimulus interval was more than 30 s. Each odour was presented two times in succession. The presentation order of odours was randomized.

In experiments with dose–response curves, 50 µL of odourant solution was directly placed in a test tube. The airflow passed through the test tube and then flowed to an empty glass pipette. We presented each odour starting with the lowest dose (5 × 10^−5^) and ending with the high dose (5 × 10^−2^) to avoid adaptation. The other procedures were the same as described above.

### Single-sensillum recordings

The methods used for SSR followed those of previous studies^16,42^. After cold anaesthesia, a mantis was immobilised ventral-side-up on an acrylic plate with dental wax. The antennae were fixed to the plate with beeswax at 1–2 mm intervals. The antennae were observed through a light microscope (Nikon AZ100). Activities of OSNs were recorded by inserting a tungsten electrode into the basal cavity of a basiconic sensillum on the antennae using a micromanipulator. The reference electrode was inserted into the base of the antennae. The tungsten electrodes were electrolytically sharpened by dipping them repeatedly into a saturated KNO_2_ solution. The room temperature was kept at 22–23 °C during the recording.

Electrical signals were processed with a preamplifier (MEZ-8301, Nihon Kohden, Tokyo, Japan), amplified with a main amplifier (EX-1; Dagan Corporation, Minneapolis, USA), and filtered at 2 kHz and 300 Hz through a high/low pass filter. Signals were digitised and recorded at a sampling rate of 10 kHz using an analogue–digital converter (Power lab 4/30, AD Instruments Japan Inc., Nagoya, Japan).

### Data analysis

We pooled data from males and females because there was no detectable difference between them. The SSR data were analysed using Spike2 software (Cambridge Electronic Design, UK). Spikes were classified into three units by template matching implemented in Spike2, and then the classification of spikes was manually corrected. We defined the response intensities of individual OSNs as the spike frequency (Hz) during 2 s after the onset of odour stimulation subtracted by that during 2 s before the onset of stimulation. The mean response intensity for each odour was used for analysis. Responses to specific odours were excluded from the analysis if we failed to record responses to the odours from most sensilla. ANOVA, hierarchical cluster analysis, heatmap, principal components analysis, and three-dimensional scatter plots were made using R 3.6.1 software^44^ with pvclust, psych, gplots, RColorBrewer, and scatterplot3d packages. Clustering was performed using Ward’s method, and the distance was defined as 1 − *r*, where *r* is the correlation coefficient between OSN responses to odours. Using the distance based on the correlation coefficient, we focused on the relative responsiveness of OSNs to odours.

## Supplementary Information

Supplementary Figures.

## Data Availability

The electrophysiological datasets used in this study are available from the corresponding author on reasonable request.
