# Supplementary material for: Aldehyde-specific responses of olfactory sensory neurons in the praying mantis
Source: Sci Rep. 2021 Jan 20;11:1856. doi: 10.1038/s41598-021-81359-5 (PMC7817670; doi:10.1038/s41598-021-81359-5)
Supplement: Supplementary file 1 — Supplementary Figures. [file 41598_2021_81359_MOESM1_ESM.pdf]

Supplementary Information

Aldehyde-specific responses of olfactory sensory neurons in the praying mantis

Kota Ezaki[1], Takashi Yamashita[1], Thomas Carle[1,2], Hidehiro Watanabe[2], Fumio Yokohari[2], and Yoshifumi Yamawaki[1]*

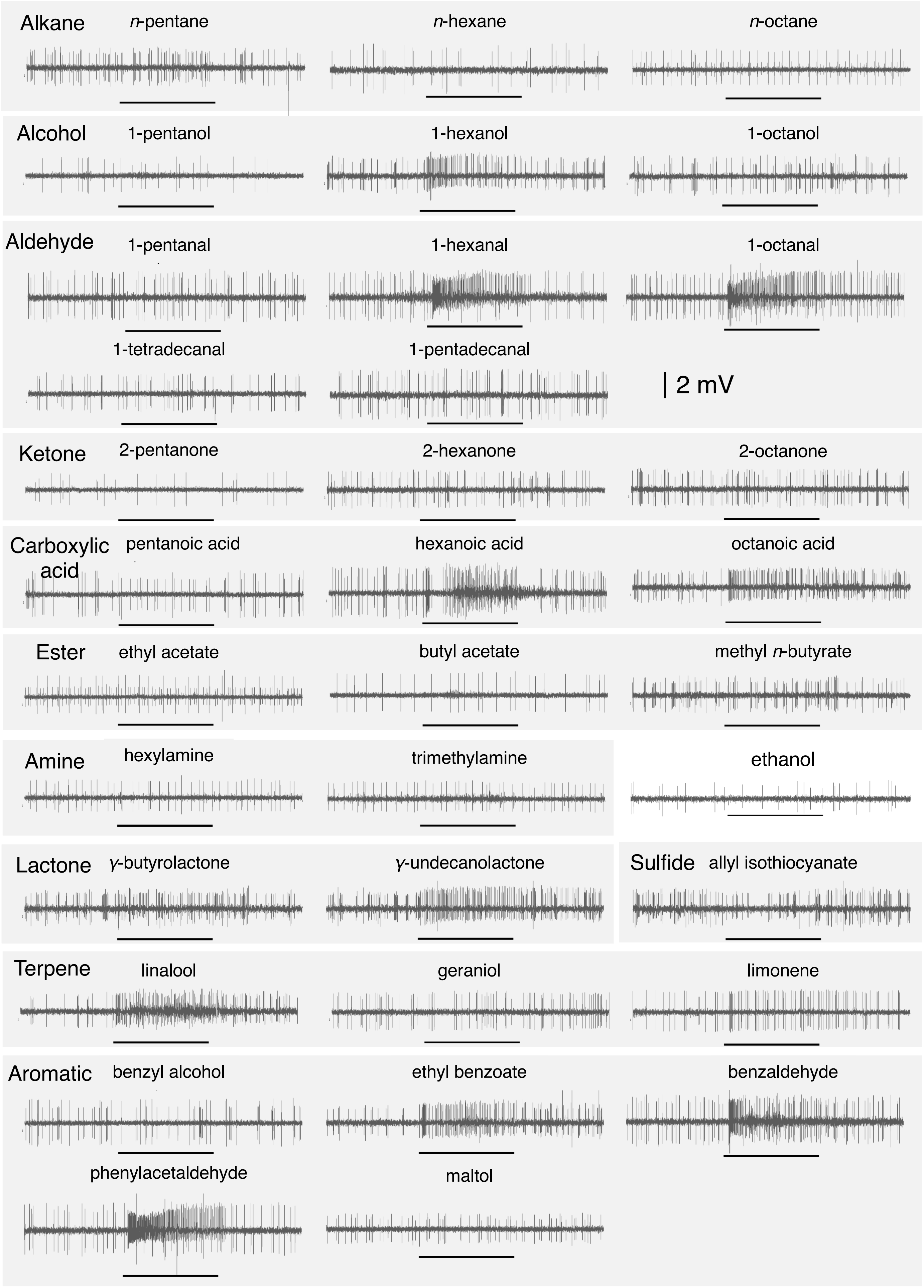

Supplementary Figure S1. Sample responses to odours and solvent (ethanol) recorded from the same basiconic sensillum. Black bars under the recordings indicate the period of odour stimulation (2 s).

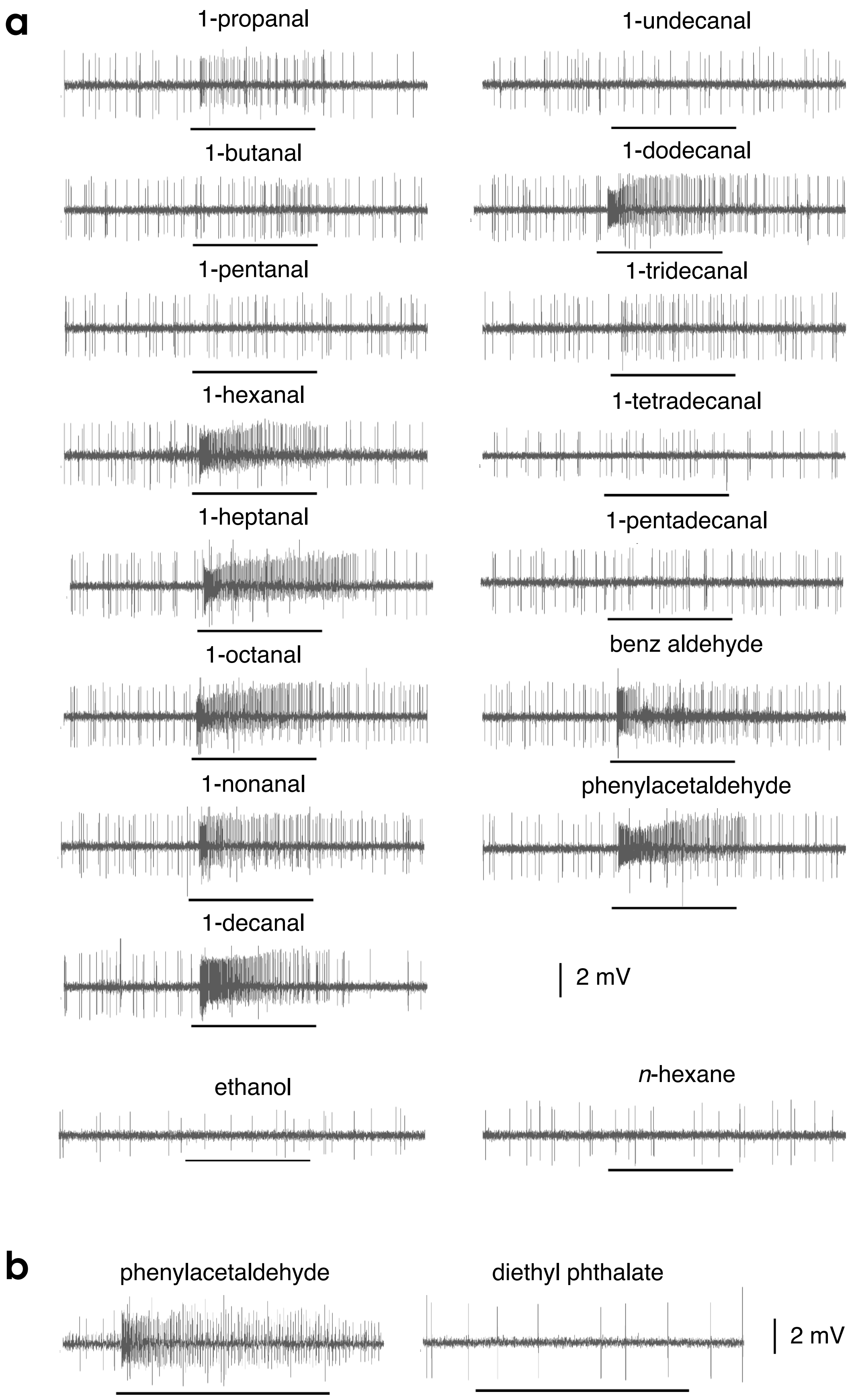

Supplementary Figure S2. **a** Sample responses to aldehydes and solvents (ethanol and n-hexane) recorded from the same basiconic sensillum. Black bars under the recordings indicate the period of odour stimulation (2 s). **b** Sample responses to phenylacetaldehyde and solvent (diethyl phthalate) recorded from another sensillum.

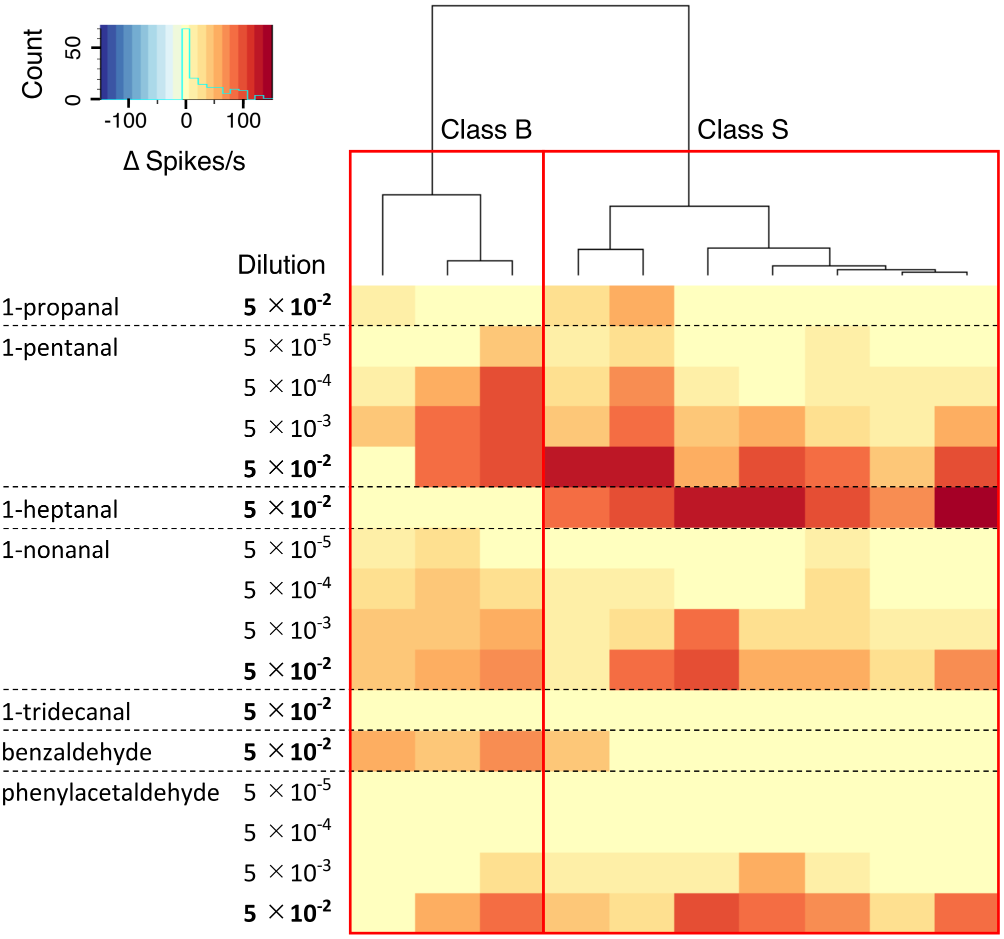

Supplementary Figure S3. A heat map of the response intensities of a single OSN to aldehydes at different dilutions. Each column indicates a single OSN showing unit 3-type spike. A dendrogram of a hierarchical cluster analysis (shown above the heat map) suggested two groups. One (n = 7, right) showed stronger responses to 1-pentanal than to 1-nonanal and weak responses to benzaldehyde, suggesting that this group belonged to class S. The other (n = 3, left) responded to 1-pentanal, 1-nonanal, and benzaldehyde at similar spike rates, suggesting that this group belonged to class B. R 3.6.1 software (https://www.r-project.org/) was used for analysis.
